# Evaluation of Disparities in Emergency Department Admission and Wait Times for Non-English Preferred Patients

**DOI:** 10.5811/westjem.21242

**Published:** 2025-05-12

**Authors:** John Wong-Castillo, Daniel Berger, Juan Carlos Montoy, Riham Alwan

**Affiliations:** * University of California San Francisco – Fresno, Department of Emergency Medicine, Fresno, California; † Virginia Commonwealth University Health System, Department of Emergency Medicine and Internal Medicine, Richmond, Virginia; ‡ University of California San Francisco, Department of Emergency Medicine, San Francisco, California

## Abstract

**Introduction:**

Patients who prefer to communicate in a language other than English are vulnerable to the consequences of medical communication barriers. Studies of non-English language preferred (NELP) and English language preferred (ELP) patients have shown differences in rates of hospital admission and wait times—factors known to be related to increased costs and lower patient satisfaction. However, few studies include languages other than Spanish or account for patient acuity level.

**Methods:**

We performed a retrospective cohort study at an urban, Level I trauma center from January–December 2020. Patients were grouped by language preference, with NELP languages grouped into three categories: Spanish; Chinese (Mandarin, Cantonese, Taishanese, Taiwanese, and Zhongshan-Chinese dialect); and other (all other remaining languages). We extracted age, sex, race, ethnicity, language preference, emergency department (ED) discharge disposition, and Emergency Severity Index Score (ESI) from the electronic health record. The primary outcome was the hospital admission rate. Secondary outcomes were the time from patient arrival to placement in the treatment room and the time from patient arrival to disposition. We analyzed data with chi-square tests, logistic, and linear regressions.

**Results:**

Of the 58,079 unique ED encounters, 26.4% (15,307) patients identified as NELP. Within NELP patient encounters, 75.0% preferred Spanish, 13.9% preferred Chinese, and 11.1% preferred another language. After adjusting for age and acuity, Spanish language- and Chinese language-preferred patients were at 16% and 14% higher odds of admission, respectively (odds ratio [OR] 1.16, 95% confidence interval [CI] 1.10–1.23 and OR 1.14, CI 1.02–1.27 respectively), compared to ELP patients. NELP patients waited an average 5.4 minutes longer to be roomed (95% CI 4.46–6.29) and 15.6 minutes longer until disposition (95% CI 12.62–18.54, *P*<0.05). This discrepancy was greater for patients triaged at lower acuities, with ESI 5 Spanish language- and Chinese language-preferred patients waiting an average of 50.3 and 90.6 minutes longer than ELP patients until disposition (95% CI 17.67–83.57; and 95% CI 24.31–81.57 respectively).

**Conclusion:**

After adjusting for acuity level and age, non-English language preferred patients were at higher odds of admission and experienced disparate wait times, especially at lower acuity levels. Further investigation is needed to understand the causes of these differences and mitigate these health inequities.

## INTRODUCTION

Communication between patients and physicians is an essential aspect of acute care in the emergency department (ED). Patients who prefer to communicate in a language other than English—non-English language preferred (NELP)—are vulnerable to the effects of medical communication barriers.^1^,^2^ In the United States, 21% of the population speaks a language other than English at home, with 9% of the population reporting that they speak English less than “very well.”^3^ These persons often belong to communities that have historically faced health disparities including lower triage priorities and greater mortality rates in the hospital.^4^ For many NELP patients, these issues manifest in the ED. Due to concerns related to factors such as cost of treatment, immigration status, and lack of consistent primary care,^5^,^6^ the ED is often NELP patients’ main point of entry into the healthcare system.^7^,^8^ Patients’ NELP status has been associated with increased admission and readmission rates for certain language groups,^4^,^9^,^10^ as well as increased likelihood to have an unplanned ED return visit within 72 hours,^11^ which results in additional costs to the healthcare system and potential inefficiencies in care.

In situations where patients and physicians cannot directly communicate in the same language, professional interpreters serve an important role. Interpreters increase accessibility and delivery of care for patients, and they provide superior patient education compared to language-discordant interactions.^12^,^13^ When professional interpreters are underused in the ED, there are lower levels of patient satisfaction, fewer diagnostic procedures ordered, and increased miscommunication of discharge information.^2^,^14^,^15^ Even when professional interpreters are used, miscommunication may persist.^16^,^17^ Given the fast-paced, acute care setting of the ED, emergency physicians face resource constraints and workload strains that force quick decisions with limited information.^18^ This combination of real and perceived time pressures may lead to bias, especially when added to the challenges of language-discordant care, defined as when a patient’s primary spoken language differs from the primary language of the clinician.^14^

While many studies have shown racial and ethnic disparities in ED care,^4^,^19^ there is limited research on operational differences in care between NELP patients and English language preferred (ELP) patients. Previous literature has shown that people of color experience longer mean wait times than White patients.^20^,^21^ However, these studies do not assess language proficiency as a barrier to timely care within these diverse groups. At the same time, admission rate differences between NELP and ELP patients remain unclear, with mixed results based on acuity^22^ or with a limited focus on Spanish-speaking patients.^23^ In this study, we investigate differences in care between NELP and ELP patients across three operational categories: hospital admission rate; time from arrival to treatment room; and time from arrival to disposition.

**Population Health Research Capsule**

What do we already know about this issue?

*Non-English language preferred (NELP) status is associated with increased admission, readmission rates, and likelihood of unplanned Emergency Department (ED) return visits within 72 hours.*

What was the research question?

*Are there operational differences in ED care for NELP patients?*

What was the major finding of the study?

*Spanish- and Chinese language[1]patients had 16% and 14% higher odds of admission, respectively, compared to ELP patients. At Emergency Severity Index 5 (ESI 5), NELP patients waited 52.9 minutes longer for disposition than English patients (P<0.05).*

How does this improve population health?

*With an increasing proportion of NELP patients presenting to EDs, more must be done to address disparities in wait times and admission rates.*

## METHODS

This retrospective cohort study uses race, ethnicity, and language (REAL) data captured from Zuckerberg San Francisco General Hospital and Trauma Center’s electronic health records (EHR) (Epic Systems Corporation, Verona, WI). 24 The policy for the collection of REAL data is outlined in San Francisco Department of Public Health guidelines.^25^,^26^ We used a report to extract parameters of interest from the EHR in bulk. The study design was reviewed and approved by University of California San Francisco Institutional Review Board (IRB). The applicable criteria of health records identified, sampling method, missing-data management plan, and IRB approval, suggested by Worster and Bledsoe,^27^ were upheld in reviewing health records.

### Setting

We conducted this study at an urban, Level I trauma center between January–December 2020.

### Language Data Collection

Per institutional policy, ED patients are asked by ED staff during check-in about their language preference for the encounter. This preference can be changed by the patient at any time during the encounter.

Patients who reported English as their preferred language used in communicating with clinicians were marked as ELP. Patients who chose any other language were considered NELP. We grouped the NELP languages into three categories: Spanish; Chinese (Mandarin, Cantonese, Taishanese, Taiwanese, and Zhongshan-Chinese dialect); and other (all other remaining languages). Spanish and Chinese categories reflect the largest ethnic groups treated at this site and the most common languages spoken at this center.

Healthcare encounters at this center were interpreted by certified bilingual clinicians and contracted interpretation services by qualified interpreters delivered through phone or video tablet. In-person interpretation services by qualified non-clinical staff were much more limited in both languages and hours available compared to the 24/7 remote contracted service. The frequency of use of each modality was not recorded.

### Variables

Data extracted from the EHR included age, sex, race, ethnicity, language preference, chief complaint, primary diagnosis, ED discharge disposition, and Emergency Severity Index Score (ESI), which was used as a proxy for acuity. Sex, race, ethnicity, and language preference were self-reported. We also extracted times, including length of stay, time from arrival to treatment room, room to clinician, and clinician to disposition.

The primary outcome was hospital admission rate. Secondary outcomes included time from patient arrival to placement in the treatment room and time from patient arrival to disposition. We defined hospital admission rate as the rate of admission to the in-patient hospital or transfer to an acute care facility. Arrival to room was defined as the time from patient registration in the ED to when they were brought to their assigned ED room. Lastly, arrival to disposition was defined as the time from registration to disposition or discharge order.

### Exclusion Criteria

Patient records that were missing an ESI score or time stamps related to time from arrival to room or arrival to disposition were not included in the analysis. We also excluded observations with any missing variables for the specific model from that model’s analysis. The number of missing records for each variable are included in Appendix A.

### Statistical Methods

All analyses were performed using R v4.3.3 (R Foundation for Statistical Computing, Vienna, Austria) and RStudio v2023.12.1+402 (RStudio PBC, Boston, MA). We used chi-square tests and Student’s t-tests to compare the demographic characteristics of NELP and ELP patients. Chi-square tests were also used to compare the percentages of ELP vs. Spanish language preference patients who were assigned as low acuity (4–5). To examine differences between NELP and ELP patients in the hospital admission outcome, we used unadjusted and adjusted logistic regression models to estimate odds ratios (OR) with 95% confidence intervals (CI). For the two continuous time-based outcomes (arrival to room and arrival to disposition), we fitted adjusted linear regression models. All analyses used single ED encounters as the unit of observation, and all adjusted analyses controlled for patient age (<18, 40–64, 65–74, 85+ vs. 19–39), language preference (Spanish, Chinese, other vs. English), and ESI/Acuity level (2–5 vs. 1).

## RESULTS

Of the 58,079 unique ED encounters in the study period, 26.4% were NELP patients (Table 1). The NELP patients were more likely to be female (47.6% vs 33.4%, *P*<0.05), slightly older (mean age 45.9 vs 45.1, *P*<0.05), and identify as non-White (81.2% vs 70.5%, *P*<0.05). Within NELP patient encounters, 75.0% preferred Spanish, 13.9% preferred Chinese, and 11.1% preferred another language (Table 1). In total, 20.7% of patients were admitted from the ED. In the unadjusted chi-square analysis, more NELP patients were admitted from the ED overall at 22.6% vs 20.0% of ELP patients admitted (Table 2) (*P*<0.05*)*.In the unadjusted regression using language preference as the single variable (Table 3, Model 1), Spanish-language preference had lower odds of admission compared to ELP patients (odds ratio [OR] 0.91, 95% confidence interval [CI] 0.86–0.96). In contrast, patients who identified Chinese or other as their preferred language had higher odds of admission compared to ELP patients (OR 2.61, 95% CI 2.39–2.59 and OR 1.72, CI 1.55–1.91, respectively).

In the multivariate model that adjusted for age and acuity level (Table 3, Model 2), NELP patients overall had greater odds of admission. However, after adjusting for age and acuity level, Spanish language- and Chinese language-preferred patients showed increased odds of admission (OR 1.16, 95% CI 1.10–1.23 and OR 1.14, CI 1.02–1.27, respectively) compared to ELP patients (*P<0.05*). Admission rate was not significantly different from ELP patients for patients who chose another language. In the chi-square analysis, there was no statistically significant difference between the proportion of English language- vs Spanish-language preferred patients who were assigned as low acuity 4–5 (*P*=0.37).

Patients who visited the ED waited an average of 23 minutes from arrival until they were roomed. Compared to ELP patients, NELP patients waited an average 5.4 minutes longer to be roomed (*P<*0.05). By category, patients who prefer Spanish, Chinese, or other languages experienced an average increased time to arrival to room of 5.9, 3.8, and 3.4 minutes (Table 3, Model 3), respectively, compared to ELP patients (*P<*0.05).

**Figure 1:**
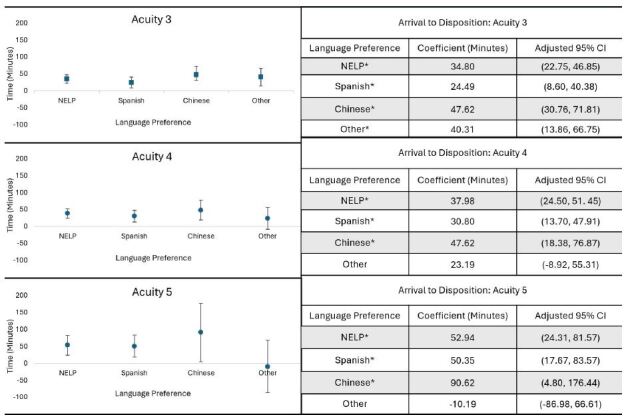
Distributions and tables of adjusted arrival to disposition times for emergency department visits of acuity levels 3, 4, and 5 of non-English-language vs English language-preferred patients, overall and by language preference. *Indicates statistically significant at a level of *P* < .05. Model adjusted for age and interaction variable created between language preference and ESI/acuity levels 1–5; reference level for language preference is ELP, ESI/acuity level is 1, and age is 19–39. *CI*, confidence interval; *NELP*, non-English language preferred; *ELP*, English language preferred.

Patients who visited the ED waited an average of 218 minutes from arrival until they received disposition. As a whole, NELP patients waited 15.6 minutes longer until disposition (*P<*0.05). Time to disposition for patients with indicated language preferences of Spanish, Chinese, and other averaged 17.2, 9.9, and 11.5 minutes longer, respectively, (Table 3, Model 3) than ELP patients (*P<*0.05; *P<*0.05, *P<*0.05). The difference in time to disposition between NELP and ELP groups was wider at lower acuity levels (3, 4, and 5) (Figure 1). At these levels, the overall average time spent was 236, 148, and 94 minutes, respectively. However, at ESI 4, Spanish language- and Chinese language-preferred patients waited an additional 30.8 and 47.6 minutes of time, respectively, to disposition compared to ELP patients (*P<*0.05). At the lowest acuity ESI 5, Spanish language preferred- and Chinese language preferred-patients waited for an average of 50.3 and 90.6 minutes more than ELP patients (*P<*0.05). Other language-preferred patients’ arrival to disposition times were not significant at ESIs 4–5.

## DISCUSSION

In this retrospective study of the association between a patient’s language preference and operational outcomes, NELP patients showed different odds of admission and treatment times compared to ELP patients. Adjusting for age and acuity level, NELP patients, as a whole, as well as those who selected Spanish and Chinese, experienced higher overall admission rates compared to ELP patients. While our findings were largely consistent, there was notable variation specific to the models.

The unadjusted regression that included language preference as the sole variable showed that Spanish language-preferred patients had lower odds of admission compared to ELP patients. When the variables of age and acuity level were accounted for in the multivariate model, Spanish language-preferred patients were at higher odds of admission compared to ELP patients. This trend seems to align with Schulson et al’s finding that patients with limited English proficiency were more likely to be admitted to the hospital than English-proficient patients for any admission.^23^ Both of our regression models showed increased odds of admission for NELP patients as a whole compared to ELP patients.

Admission decisions in the ED are informed both by the patient’s clinical condition and non-medical factors. Hunter et al examined the non-medical factors that influenced clinician disposition decisions for non-critically ill patients. They found that almost half of the admission decisions for patients in their study were influenced by factors including lack of information about the patient’s baseline condition, need for diagnostic testing, recent ED visits, and perceived inability to follow-up in a primary care setting.^28^ All these issues affect NELP patients. Previous studies have reported that non-English speakers, even within the same racial/ethnic group, have poorer access to preventative care and insurance coverage.^7^,^29^,^30^ Additionally, communication barriers that result from clinician-patient language discordance are well documented in the literature and can contribute to diagnostic uncertainty.^1^,^17^,^31^

As a percentage of its language population, Spanish language-preferred patients were admitted at a lower rate than ELP patients. One possible explanation could reflect this center’s comfort in managing Spanish-speaking patients. Spanish is the most common non-English language in this community, with both in-person and phone interpretation options available. Although this study lacks the ability to track the Spanish-speaking proficiency and certification level of all patient-facing ED staff members, given the area it serves, there are likely more Spanish-speaking clinicians in the ED than for any other non-English language. This increased availability of Spanish-speaking clinicians to conduct language-concordant encounters with Spanish-speaking patients likely reduces diagnostic uncertainty and allows for more discharges, as reflected by the lower patient admission rate.

For Spanish-speaking Hispanic populations, specifically, language-concordant interactions between physicians and patients have been associated with improved patient participation in treatment plans^32^ and improved glycemic control.^1^ Although interpreters are not direct substitutes for language concordant care,^13^ when professional interpreters are used, patients receive more health education^13^ and there is a lower likelihood of communication errors.^33^

Although there is a tendency to aggregate NELP patients as a monolithic group, patients with different language preferences are made up of different races and ethnicities with unique cultural attitudes, beliefs, and interactions with the healthcare system. Use of the ED by Spanish-speaking patients may be different compared to other language groups. Parast et al noted that Hispanic patients reported higher ED utilization and a more profound lack of access to primary care compared to White patients.^6^ Recent national utilization trends add to this finding: ED visits among Hispanic persons <65 years of age with Medicaid increased the most compared to other races/ethnicities, from 46.3% in 2011 to 62.7% in 2021.^34^ Hispanic patients who prefer Spanish may be using the ED for less severe complaints to compensate for a lack of primary care access, resulting in a lower rate of admission.

However, it is also possible that clinicians and institutions are underestimating this group’s illness severity and inappropriately discharging patients who should be admitted. A recent study by Rojas et al found that children accompanied by caregivers preferring languages other than English are more likely to be triaged as non-urgent in their pediatric ED.^35^ At the race and ethnicity level, a multistate study found that severely injured Black and Hispanic trauma patients were more likely to be undertriaged than White patients.^36^ Joseph et al found similar trends in the undertriage of patients and suggested that language barriers may be an underlying contributor to this disparity due to incomplete information gathering on time-pressured triage nurses.^37^ However, in our study we found no significant difference between English language- and Spanish language-preferred patients who were assigned acuities 4–5 (non-urgent). This may again be due to institutional comfort with this patient population, fewer pressures on triage nurses at this center, or more conservative triage designation policies.

In contrast, Chinese and other language-preferred patients were admitted more frequently in their relative populations compared to ELP patients (39.48%, *P<*0.05 and 30.10%, *P<*0.05, respectively). At this center, these NELP languages are less common than Spanish. Fewer resources or less familiarity may encourage risk-averse physicians to conservatively admit the patient, which is supported by the consistent trend in literature of NELP patients’ increased admission rates or readmissions within 30 days.^9^–^11^,^38^,^39^ Specific linguistic and cultural differences with Chinese language-preferred patients may also help explain this admissions trend compared to Spanish language-preferred patients. Asian Americans, especially those who speak a different language than English, face similar issues to Hispanic patients in lack of access to healthcare and inconsistent medical care.^5^ However, Asians as an ethnic group represent more than 100 languages/dialects compared to 99% of Hispanics with limited English proficiency who speak Spanish.^40^ This linguistic diversity is an additional source of complication for physicians and interpreter services.

Chinese language-preferred patients may also rely on alternative treatments or be more conservative in seeking Western healthcare until they are ill enough to warrant admission. Clough et al noted that Asian immigrants may hold adversarial beliefs about Eastern vs Western medicine, carry social stigma around certain diseases,^41^ and have attitudes that any form of healthcare (even preventative) should be withheld until symptoms are present.^42^ If the presenting clinical conditions of these patients are sicker than other groups, it is understandable that they are more frequently admitted.

The odds of admission were not the only difference between ELP and NELP patients, with significant increases in the times of arrival to treatment room and arrival to disposition observed within each NELP language category. Compared to differences in rooming time, NELP patients waited much longer than ELP patients to receive disposition. This suggests discrepancies in treatment time or disposition planning for NELP patients compared to ELP patients. Inadequate capture of diagnostic information during the initial exam may force uncertain physicians to rely on additional imaging^37^ and labs, thereby increasing post-room waiting times. A previous study found that Spanish-speaking and American Sign Language patients were more likely to receive imaging studies.^43^ In the literature, NELP patients have been subject to increased use of radiographs, ultrasounds,^23^ and even three times as much computed tomography for abdominal pain compared to English-proficient patients.^38^

These discrepancies were greatest at ESI 5 levels for Spanish-language and Chinese-language preferred patients. One reason for these disparities could be due to the nature of emergency medicine. In the ED, high-acuity and life-threatening presentations are evaluated urgently with resource-intensive algorithmic treatments.^44^ In contrast, low-acuity complaints are less time-sensitive but may require extensive interviewing or other time-consuming resources. Although some of the increased length of stay could be warranted from additional time spent interviewing patients with interpreters or waiting for an interpreter to become available to provide the best care, it is unlikely that the additional 30–90 minutes that these lower acuity NELP patients waited prior to disposition could be explained entirely by ideal interpreter use. It is also possible that low-acuity patients may experience the worst versions of both physician interactions and waiting times in the ED. Saunders noted that patients in his study assigned the lowest acuities experienced the longest times simply moving through the ED yet also had brief evaluations and treatments.^44^ These logistical differences could affect clinical outcomes.

Guttman et al findings show that a longer mean length of stay is associated with an increased risk of admission or short-term mortality in low-acuity patients.^45^ As a result, the time disparities for low-acuity NELP patients represent not only an equity and satisfaction issue but also a potential hazard for non-urgent and emergent patients, with Luo et al contending that the resources required to treat low-acuity patients are not negligible and even may delay care for higher acuity patients.^46^ These results reflect stark differences in patient experience that could become clinically significant or, at the very least, negatively affect patient satisfaction.^47^,^48^

In caring for NELP patients, clinicians face additional time-related challenges including obtaining the appropriate translator and waiting for them to interpret through the history-taking and counseling their patients. Ramirez et al reported that these perceived increased physician time requirements even serve as a barrier to interpreter usage in the first place.^14^ However, when non-English speaking patients received inadequate counseling, they misunderstood discharge instructions, including how to manage their condition, take their medications, recognize symptoms that should prompt a return to care, and know when to follow up.^49^,^50^

To an extent, clinicians may be justified in their concerns, as Fagan et al found that compared to patients not requiring an interpreter, patients using a telephone interpreter had significantly longer mean clinician times.^51^ However, this difference was not appreciated with in-person interpreter services,^51^ which suggests a possible solution to help mitigate this health inequity is to expand the availability of in-person services or improve existing telephone-facilitated encounters. At this study’s site, telephone interpretation was by far the most common option and may be a contributor to the time disparities in disposition for NELP patients. Thus, due to real and perceived concerns over additional effort and time costs, clinicians may have been more reluctant to see low-acuity NELP patients or take longer to determine patient disposition.

## LIMITATIONS

Limitations of this study include its single-center design, with institutional resources and practices potentially limiting the generalizability of the results. Lower-resourced centers, centers with less familiarity with NELP populations, or centers with more clinician-patient language concordance, would be expected to have different results. In addition, the modality of interpretation services used for these encounters (in-person clinician, non-clinician, remote contracted service) was not recorded. There is a possibility that extremes in length of stay could be due to one particular modality. We also did not control for sex, leaving it as a potential confounder. Future research should control for this variable or explore whether there are intragroup differences between sexes in NELP populations in their experiences of care.

While the most common NELP languages at this ED were grouped into Spanish and Chinese, other sites may have a different demographic makeup and feature different non-English patient languages with unique challenges and results. For statistical purposes, this study’s categorization of Chinese as a language preference was the combination of many dialects. Dialects like Mandarin and Cantonese are more popular globally and, thus, interpretation services are more readily available for these dialects than one such as Taoshinese, potentially influencing physician comfort and time-based outcomes. Furthermore, inappropriate designations of NELP or ELP status, specifically inaccuracies in the EHR or inaccurate self-reporting would likely over- or underestimate the number of NELP encounters. For example, a patient may be more proficient in a non-English language but still choose English as the preferred language of their encounter. Lastly, the observational nature of this study did not lend to identifying the causes of the discussed disparities. Further studies are needed to investigate possible solutions to this health inequity.

## CONCLUSION

Emergency Department patients who preferred to communicate in a language other than English were at higher odds of admission and had disparities in wait times that were especially pronounced at lower acuity levels. To improve the efficiency of ED operations and promote health equity, further investigation is needed to identify the causes of these differences, evaluate existing language interpretation options, and encourage centers to review practices that may impact the care of NELP patients. Centers should begin this investigation with the collection of accurate language interpretation data. These metrics are necessary to assess ED throughput and gauge responsiveness to the specific communities that are served.

## Supplementary Information



## Figures and Tables

**Table 1:** Sample characteristics by non-English language preferred (NELP) and English language preferred patients (ELP). (Total of 58,079 Encounters)

Variable	NELP	ELP	*P*-value^*^

	n=15,307 (26.4%)	n=42,682 (73.6%)	
Sex			<0.05
Female	47.6%	33.4%	
Male	52.4%	66.5%	
Age Category			<0.05
0–18	13.1%	4.2%	
19–39	31.2%	38.8%	
40–64	36.3%	45.3%	
65–74	9.5%	8.0%	
75–84	5.2%	2.2%	
85+	4.1%	1.3%	
Race/Ethnicity			<0.05
American Indian or Alaska Native	0.05%	1.0%	
Asian	18.4%	7.5%	
Black Or African American	0.5%	32.1%	
Native Hawaiian or Other Pacific Islander	0.3%	2.0%	
Other	3.5%	5.6%	
White	2.1%	29.5%	
Yes - Hispanic, Latino/A, Or Spanish Origin	72.3%	19.2%	
Blank	2.8%	2.5%	
Decline To Answer	0.1%	0.4%	
Language Preference			
English	-	100%	
Spanish	75.0%	-	
Chinese	13.9%	-	
Other	11.1%	-	
Emergency Severity Index/Acuity			<0.05
1	5.5%	7.1%	
2	20.3%	25.5%	
3	54.7%	46.1%	
4	18.3%	18.6%	
5	1.2%	2.8%	

*P*<0.05 indicates differences between ELP and NELP patients within the subgroup.

*NELP*, non-English language preferred; *ELP*, English language preferred.

**Table 2:** Admissions data of English language preferred patients (ELP) and non-English language preferred patients (NELP) overall and by language preference (Spanish, Chinese, and Other).

Category	Percentage Admission	*P*-value*
Total	20.7%	-
English Language Preferred	20.0%	-
Non-English Language Preferred	22.6%	<0.05
Spanish (as % of all Spanish Language patients)	18.5%	<0.05
Chinese (as % of all Chinese Language patients)	39.5%	<0.05
Other (as % of all Other Language patients)	30.1%	<0.05

* *P*<0.05 indicates differences between ELP compared to NELP overall and the NELP subgroups.

*ELP*, English language preferred; *NELP*, non-English language preferred.

**Table 3:** Differences in likelihood of hospital admission and average times for arrival to room and arrival to disposition for patients with non-English vs. English language preference, overall and by NELP category.

	Likelihood of Hospital Admission: Model 1*		Likelihood of Hospital Admission: Model 2‡	

Language	Odds Ratio	95% CI	Odds Ratio	95% CI
NELP	**1.17**	**(1.12, 1.23)**	**1.15**	**(1.093, 1.213)**
Spanish	**0.90**	**(0.859, 0.955)**	**1.16**	**(1.095, 1.233)**
Chinese	**2.61**	**(2.386, 2.857)**	**1.14**	(1.023, 1.271)
Other	**1.72**	**(1.548, 1.914)**	1.11	(0.981, 1.257)

	Time from Arrival to Room: Model 3Φ		Time from Arrival to Disposition: Model 3Φ	

Language	Coefficient (Minutes)	95% CI	Coefficient (Minutes)	

NELP	**5.38**	**(4.464**–**6.288)**	**15.58**	**(12.62**–**18.54)**
Spanish	**5.93**	**(4.916**–**6.950)**	**17.21**	**(13.91**–**20.52)**
Chinese	**3.88**	**(1.732**–**6.021)**	**9.90**	**(2.941**–**16.85)**
Other	**3.46**	**(1.113**–**5.802)**	**11.55**	**(3.942**–**19.15)**

Bold indicates statistically significant at a level of *P* < 0.05.

* Model 1 compares NELP patients, overall and by NELP category to ELP patients, unadjusted for covariates.

‡ Model 2 compares NELP patients overall and by language category to ELP patients after adjusting for age and ESI/acuity level.

Φ Model 3 compares NELP patients overall and by NELP category to ELP patients after adjusting for age and ESI/acuity level.

*CI*, confidence interval; *NELP*, non-English language preferred; *ELP*, English language preferred.
